# Constraint Release for Reptating Filaments in Semiflexible Networks Depends on Background Fluctuations

**DOI:** 10.3390/polym14040707

**Published:** 2022-02-12

**Authors:** Tina Händler, Cary Tutmarc, Jessica S. Freitag, David M. Smith, Jörg Schnauß

**Affiliations:** 1 Peter Debye Institute for Soft Matter Physics, University of Leipzig, Linnéstraße 5, 04103 Leipzig, Germany; tina.haendler@uni-leipzig.de (T.H.); cary.tutmarc@physik.uni-leipzig.de (C.T.); david.smith@izi.fraunhofer.de (D.M.S.); 2 Fraunhofer Institute for Cell Therapy and Immunology, Perlickstraße 1, 04103 Leipzig, Germany; jessica.freitag@izi.fraunhofer.de; 3 Institute of Clinical Immunology, University of Leipzig Medical Faculty, 04103 Leipzig, Germany; 4 Dhirubhai Ambani Institute of Information and Communication Technology, Gandhinagar 382 007, India; 5 Unconventional Computing Laboratory, Department of Computer Science, University of the West of England, Bristol BS16 1QY, UK

**Keywords:** polymer network, constraint release, reptation, semiflexible polymer

## Abstract

Entangled semiflexible polymer networks are usually described by the tube model, although this concept has not been able to explain all experimental observations. One of its major shortcomings is neglecting the thermal fluctuations of the polymers surrounding the examined test filament, such that disentanglement effects are not captured. In this study, we present experimental evidence that correlated constraint release which has been predicted theoretically occurs in entangled, but not in crosslinked semiflexible polymer networks. By tracking single semiflexible DNA nanotubes embedded both in entangled and crosslinked F-actin networks, we observed different reptation dynamics in both systems, emphasizing the need for a revision of the classical tube theory for entangled polymer solutions.

## 1. Introduction

**Figure 1:**
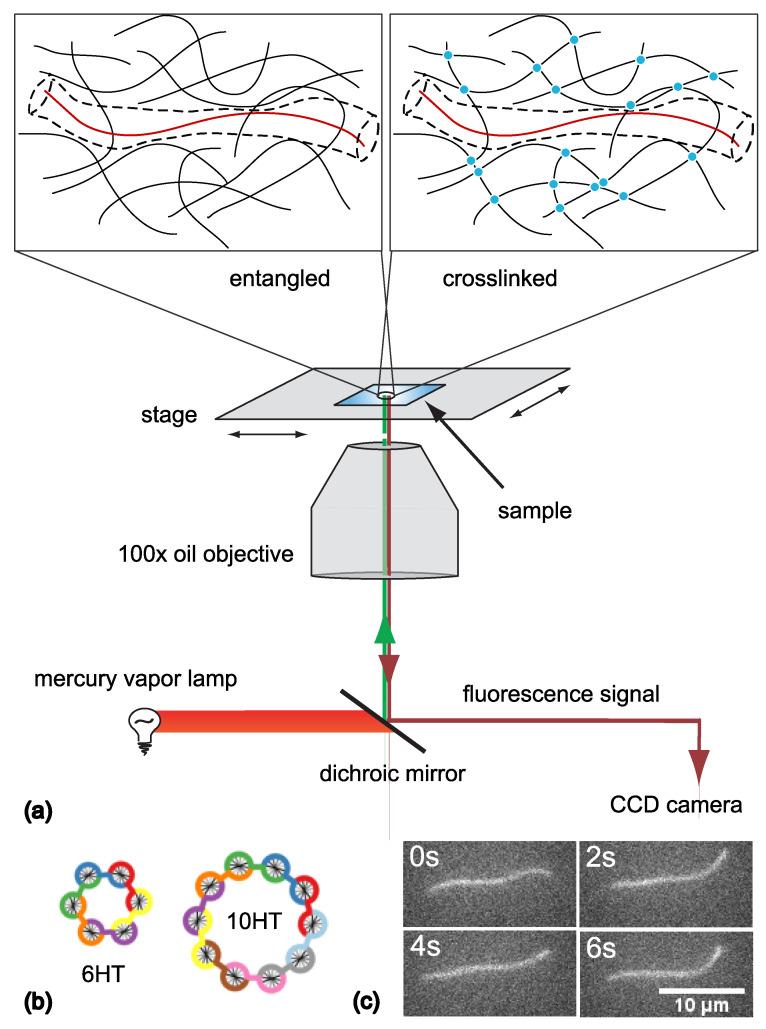
(**a**) Depiction of the experimental setup with a custom-made sample chamber. The two schematic magnifications illustrate an entangled (left) or crosslinked (right) semiflexible polymer network with an embedded tracer filament (red). The surrounding filaments (black) are either intertwined or connected by a crosslinker (blue). The dashed outline indicates the space available to the tracer filament, the so-called reptation tube. Panel (**b**) displays schematic cross-sections of the two DNA nanotube tracers with the lowest (6HT) and highest (10HT) persistence length values used in this study [17]. Each color represents a single DNA strand that builds up the DNA structure (figure adapted from Schuldt et al. [16]). Exemplary microscopy images of a single fluorescently labeled nanotube (8HT) embedded in a crosslinked F-actin network (unlabeled) are shown in panel (**c**) for four different frames, but with the same cropped image section.

Although solutions of semiflexible polymers have been studied for decades and are important in soft matter physics, biology and material science, there is still no theoretical model that sufficiently explains their unique properties [1,2]. The most successful approach is an extrapolation of the so-called tube model, which was originally developed to describe solutions of flexible polymers [3,4]. It reduces the many-body problem to a few degrees of freedom by investigating a test filament which is constrained in its motion by all other polymers of the network [1,3,4,5,6,7,8,9], see Figure 1a. Theoretical descriptions often refer to the surrounding polymer network as a static matrix [1,6,7,8,9,10], while experimental studies focus either on the reptation of actin filaments in entangled F-actin solutions [11,12,13] or on the dynamics of stiff rods in a fixed matrix [14]. Only recently, computer simulations have been employed to investigate the effects of non-static surrounding polymers [15], with results matching previous theoretical efforts [9]. Since the standard model system for semiflexible polymers is filamenteous actin, central aspects of established theories have been experimentally inaccessible [2,16,17]. By employing novel tools such as synthetic semiflexible polymer structures built from DNA [18], advanced investigations have been possible, often leading to new insights and showing discrepancies between theory and experiments [2,16,17,19]. Furthermore, a recent Brownian dynamics study investigated the effects of constraint release mechanisms in fluid and frozen semiflexible polymer networks and predicted different dynamics of test filaments within these solutions [20]. With the recently established approach to study polymer physics by using DNA nanotechnology [16,17,19], we were able to investigate the proposed differences in constraint release. As test filaments, we used semiflexible DNA nanotubes and tracked their motion in entangled—resembling fluid—or crosslinked—resembling frozen—F-actin networks, see Figure 1a.

The DNA nanotubes are very similar to F-actin filaments in thickness and length [21]. In contrast to F-actin, they can be programmed to vary in persistence length [16,17,21,22], so that measurements probing this defining property of semiflexible polymers are possible. The programmability of the so-called *n*-helix tubes (*n*HTs) arises from their special construction, see Section 2.2. We selected five types of DNA nanotubes with a persistence length range of 3 μm to 13 μm, varying around the value for F-actin which is approximately 9 μm [23].

To mimick frozen semiflexible polymer networks, we employed DNA-based actin crosslinkers [24]. They transiently crosslink actin and have been thoroughly characterized not to affect the geometry of F-actin networks at the chosen concentration [17,24], see Section 2.1. We refrained from using naturally occurring actin crosslinkers such as alpha-actinin since they have shown unspecific interactions with the fluorescent *n*HT tracers, rendering them inappropriate for this study [17,24]. The synthetic actin crosslinkers are well-defined regarding their binding strength, exclusive binding to actin structures, and their influence on actin network morphology. With this experimental system, we found differences in the dynamics of embedded tracers in entangled and crosslinked F-actin networks that verify the predicted effects of correlated constraint release in non-static polymer solutions [20].

## 2. Materials and Methods

### 2.1. F-Actin and Actin Crosslinker wLX

G-actin was prepared from rabbit muscle as described previously by Humphrey et al. [25] and Smith et al. [26] and refined by Gentry et al. [27]. For the formation of F-actin, polymerization was initiated by the addition of 10 times concentrated F-Buffer ( 1 M KCl, 10 mM MgCl_2_, 2 mM ATP, 10 mM DTT, 20 mM sodium phosphate, pH 7.5) in the sample preparation process. Networks formed from entangled actin filaments were used as an approach to the moving semiflexible polymer networks described by Lang and Frey [20]. To mimick frozen F-actin networks, actin filaments were crosslinked using the synthetic weak LifeAct^®^-based crosslinker (wLX) presented by Lorenz et al. [24]. The DNA-based wLX comprises a double DNA strand of 60 base pairs, with an actin-binding peptide (LifeAct^®^, Peptide Specialty Laboratories GmbH, Heidelberg, Germany) connected to each side a copper-free click-chemistry approach [24]. The binding dynamics of the transient crosslinker wLX resemble those of the natural actin crosslinker alpha-actinin, but wLX does not unspecifically bind to the DNA nanotube tracers [17]. In all crosslinked F-actin networks, wLX molecules were added to a ratio of 1 per 150 G-actin molecules so that the networks were fully crosslinked, but the filaments were not bundled [17,24].

### 2.2. DNA Nanotubes as Tracers

DNA nanotubes were used as semiflexible tracer filaments [21]. As described previously [17], they were hybridised from *n* partially complementary oligonucleotides (purchased from biomers.net GmbH, Germany) to form *n*-helix tubes (*n*HTs) within a thermocycler (TProfessional Standard PCR Thermocycler, Core Life Sciences Inc., Dallas, TX, USA). The *n* oligonucleotides form *n*-helix tubes (*n*HTs), see Figure 1b. One of the oligonucleotides was modified to have the dye Cy-3 attached [16,17] so that the tracer filaments could be observed with a fluorescence microscope. By changing the number of oligonucleotides from 6 to 10, the persistence length of the *n*HTs was varied from approx. 3 μm to 13 μm [16,17,21,22]. DNA nanotubes are stable for weeks after hybdridization and have been used as tracer filaments embedded in entangled and crosslinked F-actin networks before [16,17].

### 2.3. Sample Preparation and Measurement

Since the *n*HT tracers maintain their structure after hybridization if they are kept below the melting temperature of approximately 60 ${}^{\circ }C$ [21], we were able to polymerize the F-actin networks around the DNA nanotubes, thus ensuring their homogeneous distribution. For sample preparation, pre-hybridized, fluorescently labeled *n*HTs were diluted step by step and mixed with freshly thawed G-actin. For each sample, only one type of *n*HT was used. For crosslinked F-actin networks, wLX was added to the solution to a molar ratio of 150 actin monomers to 1 crosslinker molecule. Actin polymerization was initiated by the addition of 10× concentrated F-buffer so that the actin content of the final solution was 0.5 mg/mL. The final sample solutions were kept at 37 ${}^{\circ }C$ for 1h to 2h so that actin filaments were able to form around the DNA tracers. Afterwards, the sample solutions were gently placed between two glass slides previously coated with 5% bovine serum albumin or Sigmacote (Sigma-Aldrich, St. Louis, MI, USA) to prevent the networks from sticking to the glass surface. The samples were sealed with grease to avoid evaporation and left to settle for 30 min–60 min to equilibrate at room temperature before measurements were started. The measurements were also performed at room temperature. Each sample was scanned for single *n*HT filaments that were suitable for investigation, i.e., fully visible in the focal plane of the microscope and not attached or close to the sample chamber walls. The fluorescent signal of *n*HT tracers in unlabeled F-actin networks was tracked for 60s to 120s and recorded via an epi-fluorescence microscope (Leica DM-IRB, 100× oil objective, NA 1.35) at a frame rate of 10 Hz employing an attached CCD camera (Andor, iXon DV887), see Figure 1a for a schematic setup and Figure 1c for examples of recorded frames.

### 2.4. Data Analysis

**Figure 2:**
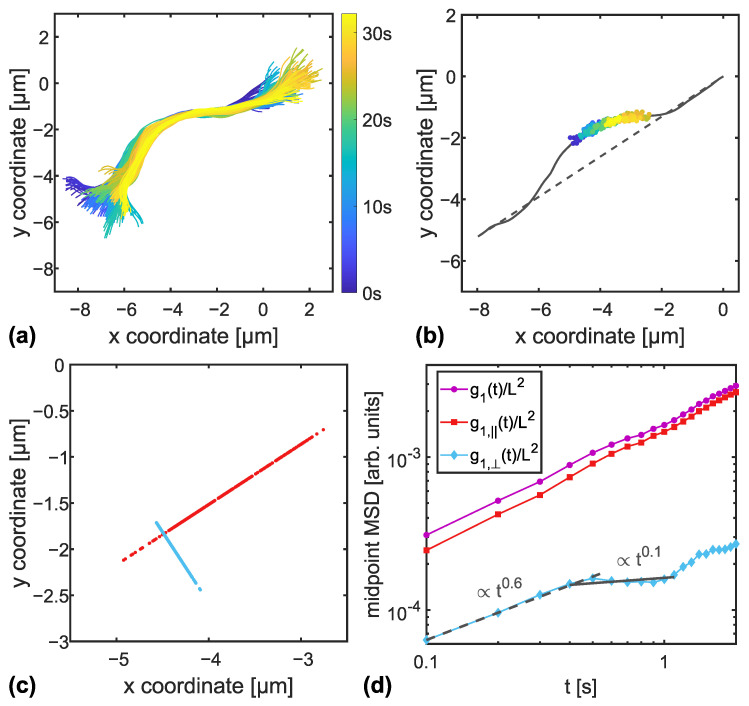
Overview of the mean squared displacement (MSD) analysis performed on an exemplary 8HT reptating in a crosslinked F-actin network. Panel (**a**) shows an overlay of filament conformations of successive frames, whereas the color code represents the corresponding measurement time. In panel (**b**), the corresponding trajectory of the filament’s midpoint is plotted together with the initial frame’s filament configuration (grey line) and the respective end-to-end line (dashed grey line). The trajectory of the midpoint is projected parallel and perpendicular to the end-to-end direction, the resulting projected trajectories are presented in panel (**c**). From the two-dimensional trajectory as well as from the projected trajectories, we calculated the MSD using Equations (1)–(3). In accordance with the plots in Ref. [20], we rescaled the MSDs with the squared contour length of the individual filament, see panel (**d**). The dashed and solid lines indicate the two power laws fitted as explained in the text.

For each measured filament, the series of fluorescent images was analysed using ImageJ (https://imagej.nih.gov/ij, accessed on 7 February 2022). First, the brightness and contrast of all images was adjusted with the plugin Stack Contrast Adjustment [28]. Then the filament conformations of the resulting images were extracted using the plugin JFilament [29]. The analysis of filament coordinates was performed employing custom-made MATLAB (The MathWorks, Natick, MA) code. In the first step, a set of subsequent frames with constant filament contour length (variation less than 5%) was chosen for further analysis. This is crucial because the calculation of the filament’s midpoint depends on the correct tracking of the filament’s end points. If an end point’s position varies too much due to bleaching of the fluorescent dye or fluctuation of the filament’s ends out of the focal plane, the midpoint’s trajectory and the resulting mean squared displacement (MSD) would give false results. Filaments with less than 60 subsequent frames of nearly constant contour length were discarded, so that the minimal evaluation time for the MSD analysis was 1.5 s [30], which is expected to include two discernible phases of reptational relaxation [8,12]. An exemplary frame series is shown in Figure 2a, where filament conformations are superimposed. For the chosen sets of frames, we calculated the coordinates of the filament’s midpoint as the middle point of each frame’s conformation. An example trajectory is plotted in Figure 2b.

As proposed by Lang and Frey [20], the projection of the midpoint trajectory was calculated parallel and perpendicular to the line connecting the two end points of the first frame’s conformation, see Figure 2b,c. From this, the MSD of the midpoint and its projections was determined by

(1) $$\begin{array}{c} g_{1}\left(t\right):=\langle {\left[\left({\vec{r}}_{N/2}\left(t\right)-{\vec{r}}_{N/2}\left(0\right)\right)\right]}^{2}\rangle \end{array}$$

(2) $$\begin{array}{c} g_{1,||}\left(t\right):=\langle {\left[\left({\vec{r}}_{N/2}\left(t\right)-{\vec{r}}_{N/2}\left(0\right)\right)\;\cdot \;\vec{e}\left(0\right)\right]}^{2}\rangle \;, \end{array}$$

(3) $$\begin{array}{c} g_{1,⊥}\left(t\right):=\langle {\left[{\vec{r}}_{N/2}\left(t\right)-{\vec{r}}_{N/2}\left(0\right)\right]}^{2}\rangle -g_{1,||}\left(t\right), \end{array}$$

where $\vec{e}\left(0\right)$ is the direction of the initial end-to-end vector [20]. The two-dimensional MSD of the midpoint as well as the MSD of the transverse and longitudinal projections ($g_{1,⊥}\left(t\right)$ and $g_{1,||}\left(t\right)$, respectively) are plotted together for an example filament in Figure 2d. All MSDs were divided by the squared contour length to avoid scaling effects stemming from tracer polydispersity [20].

To evaluate the reptation behavior of DNA nanotubes embedded in crosslinked and entangled F-actin networks, we determined the exponents of the power laws describing $g_{1,⊥}\left(t\right)$ in the first two discernible phases of relaxation. In a user-defined region where two power laws are to be expected, an algorithm automatically fitted all possible combinations of two power laws and then searched for the most probable solution by minimizing the sum of the corresponding fit residuals. An exemplary fit result is shown in Figure 2d. The crossover time from the first power law to the second was also recorded.

### 2.5. Significance Test/Wilcoxon Rank-Sum Test

To check whether there is a significant slowing down in transverse fluctuations of the polymer, we compared the exponents of the power laws describing $g_{1,⊥}\left(t\right)$ in the two determined phases of relaxation. The Wilcoxon rank-sum test was used to decide whether the two sets of exponents obtained for each *n*HT in one of the two background networks have an equal median. Below a 5% significance level, the hypothesis was rejected and we concluded that there was a significant change in power law exponents.

## 3. Results

DNA nanotubes have been reported to be useful in determining geometric properties of F-actin networks by analysing sequences of single filament configurations where the tracer filaments stayed in the reptation tube [17]. Here, we present results with the same experimental system, but a different analytical approach. We searched for subsequent microscopy frames of tracer filaments with nearly constant contour length (see also Section 2.4) enabling us to examine reptation dynamics of the filaments’ midpoint, specifically of the transverse fluctuations. From this analysis, we were able to verify differences in reptation behavior of semiflexible DNA tracers in entangled and crosslinked F-actin networks; providing experimental verification for the predicted correlated constraint release in fluid semiflexible polymer networks, which is not expected in frozen semiflexible polymer networks [20].

### 3.1. MSD of Transverse Fluctuations

For a fluid network, Lang and Frey [20] predicted a correlated constraint release compared to a fixed network, resulting in slowed down dynamical behavior for tracers in fixed surroundings. As an observable, they suggested to evaluate the MSD of the transverse fluctuations of the centre monomer of the tracer filament, $g_{1,⊥}\left(t\right)$. We analyzed the reptation of five different types of DNA nanotubes, with persistence lengths ranging from approximately 3 μm to 13 μm, each embedded in entangled and crosslinked F-actin networks of the same monomer concentration. For each filament, we calculated $g_{1,⊥}\left(t\right)$ and determined the exponents of the two first discernible power laws. These refer to two phases of relaxation by reptation. The proposed correlated constraint release is expected only in entangled networks [20], with crosslinked networks having much slower dynamics in the meantime. This should result in a stronger decrease in power law exponents from the first to the second relaxation phase for crosslinked networks.

**Figure 3:**
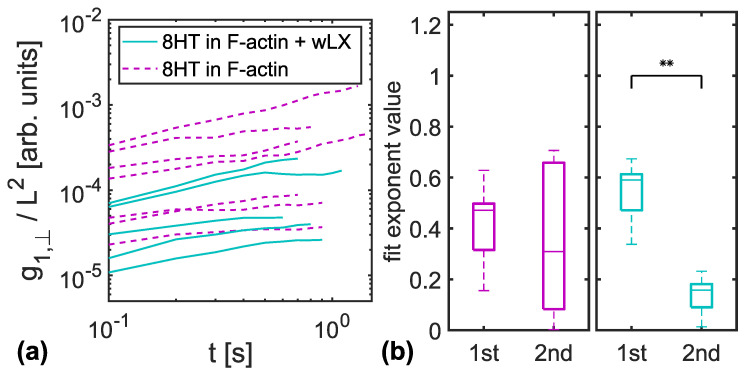
(**a**) All evaluated MSD curves of transverse fluctuations rescaled with the individual contour lengths are presented for 8HTs embedded in crosslinked (blue, solid lines) and entangled (purple, dashed lines) F-actin networks. (**b**) The boxplots show the exponents of the two distinct power laws that can be fitted to the MSDs of 8HT embedded in entangled F-actin networks (left, purple) and crosslinked F-actin networks (right, blue). The second relaxation phase exhibits significantly decreased power law exponents for crosslinked networks (** indicates below 1% significance level). This is not the case for entangled networks. In Figure 4, the corresponding results for 6HT, 7HT, 9HT and 10HT tracers are presented.

**Figure 4:**
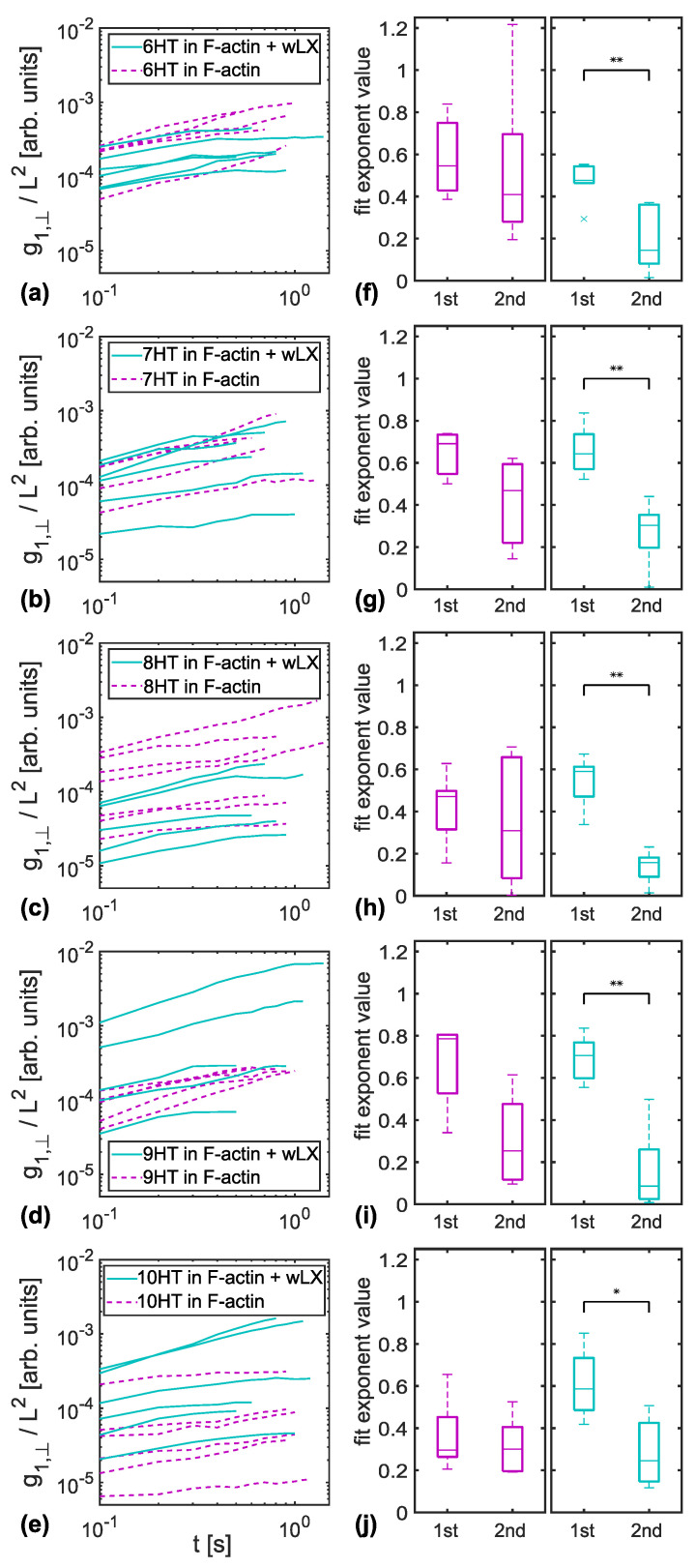
Analysed MSD curves of transverse flucuations $g_{1,⊥}\left(t\right)$ for (**a**) 6HT, (**b**) 7HT, (**c**) 8HT, (**d**) 9HT, and (**e**) 10HT tracers embedded in crosslinked (blue, solid lines) and entangled (purple, dashed lines) F-actin networks. The corresponding boxplots (panels (**f**–**j**) next to the MSD plots) summarize the exponents of the two fitted power laws as described in Section 2.4. For all tracer types, there is a significant difference between the two power law exponents for crosslinked (blue), but no such difference for entangled (purple) F-actin networks (* indicates below 5% significance level, ** indicates below 1% significance level).

For 8HTs as tracer filaments, Figure 3a shows all $g_{1,⊥}\left(t\right)$ curves in entangled F-actin (purple, dashed lines) and in F-actin crosslinked with wLX (blue, solid lines) up to the time where the second power law fit ended. We have chosen 8HT for representation due to the comparable persistence length to F-actin [17,23]. Respective results for tracers with different persistence lengths are presented in Figure 4a–e.

Panel (**b**) of Figure 3 summarizes the obtained exponents for the MSD curves presented in panel (**a**) in form of a box plot. For 8HTs reptating in a crosslinked F-actin network (blue), we found a significant decrease in power law exponents, while only a slight variation could be detected for entangled F-actin solutions (purple). The same results were obtained for all other types of DNA nanotubes used as tracers, with no apparent dependency on tracer persistence length, see Figure 4f–j. For better comparability, the data for 8HT tracers have been included in Figure 4 although they are already given in Figure 3.

Significant differences in the exponents of the first two discernible phases of relaxation were found for all crosslinked F-actin networks. It was not possible to investigate further relaxation phases by monitoring reptation due to experimental limitations. These mainly comprise bleaching of the fluorescent dye and fluctuation of the tracers in three dimensions. Both led to an apparent altered tracer contour length and, subsequently, to errors in the determination of the midpoint trajectory that is crucial for the relaxation analysis, see also Section 2.4.

### 3.2. Entanglement Times

**Figure 5:**
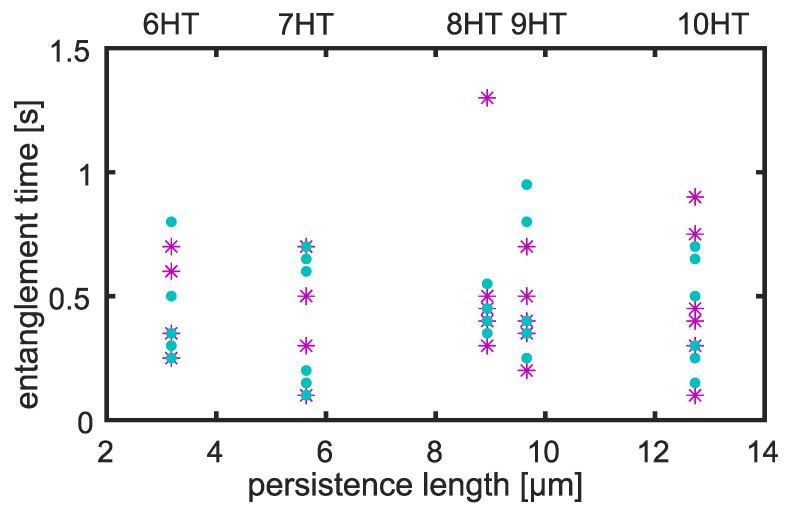
The entanglement times as determined from the crossover between two distinct phases of relaxation neither show a clear trend with persistence length of the tracer filament nor differ depending on the background network (blue circles indicate crosslinked F-actin, purple stars mark entangled F-actin). The respective helix tube type is specified in the upper *x*-axis.

The crossover time between the two observed power law regimes constituting $g_{1,⊥}\left(t\right)$ may be interpreted as the entanglement time [8,10,20]. In Figure 5, we show the resulting individual entanglement times $\tau_{e}$ for all evaluated tracer filaments. Since we used tracers of different persistence lengths $l_{p}$, we plotted $\tau_{e}$ against $l_{p}$ to investigate the theoretical prediction of $\tau_{e}\propto l_{p}^{-1/5}$ [8,15]. There is no apparent dependency on tracer persistence length and the crossover times did not differ between entangled and crosslinked F-actin background networks, but match the previously estimated order of magnitude [8].

## 4. Discussion

In this work, we compared the reptation of single semiflexible tracers embedded in entangled and crosslinked F-actin networks. Thereby, we could assess the interactions of a test filament and its surrounding network in the framework of the tube model.

### 4.1. Constraint Release in Entangled F-Actin Networks

With our experimental system, we were able to examine the prediction about different constraint release mechanisms in crosslinked and entangled semiflexible polymer networks made by Lang and Frey [20]. We verified the proposed differences in relaxation behavior by monitoring the dynamics of fluorescently labeled tracers embedded in F-actin networks and showed that these results are independent of tracer persistence length. The results suggest that the correlated dynamics of a test polymer and its surrounding filaments is the defining constraint release mechanism in entangled semiflexible polymer networks as predicted by [20]. We found a relaxation behavior with two distinct regimes for the transverse fluctuations of tracer filaments in crosslinked F-actin networks, but not in entangled F-actin solutions. The slower dynamics in the second relaxation regime indicate a persistent reptation tube in crosslinked networks [20]. In addition, the median values of the power law exponents match other theoretical predictions [10,20]. For semiflexible filaments in the presence of fixed obstacles, Nam et al. [10] calculated $g_{1,⊥}\left(t\right)\propto t^{3/4}\;\mathrm{for}\;t<\tau_{e}$ and $g_{1,⊥}\left(t\right)\propto t^{0}\;\mathrm{for}\;\tau_{e}<t<\tau_{r}$, where $t<\tau_{e}$ is the entanglement time and $\tau_{r}$ denotes the internal relaxation time.

### 4.2. Entanglement Time Dependency on Tracer Persistence Length

By monitoring the MSD of transverse fluctuations of DNA nanotube tracers in F-actin networks, we found power law exponents matching those proposed by previous studies [10,20]. In accordance with these analyses, we determined the crossover time between the two regimes to be the entanglement time. We did not find a dependency between entanglement time and tracer persistence length $l_{p}$, which was proposed to be $\tau_{e}\propto l_{p}^{-1/5}$ [8,15]. It may be possible that this dependency is concealed by other effects, such as varying tracer contour lengths. We speculate this might be resolved by measurements with a better time resolution or larger sample number. Additionally, the entanglement times we measured did not vary between entangled and crosslinked F-actin network. This is not surprising since the entanglement time is defined as the time where the reptating filament comes into contact with the surrounding filaments. This should only be affected by the geometry of the networks, which has been shown to be comparable for entangled and crosslinked F-actin networks under the same experimental conditions used in this study [17]. The numerical values of the determined entanglement times match those of previous estimations [8].

### 4.3. Conclusions

We were able to prove experimentally the different constraint release mechanisms in entangled and crosslinked semiflexible polymer networks. Together with a number of discrepancies between theory and experiments regarding the viscoelastic response of semiflexible polymer networks [2], this encourages an expansion of the established tube model theories and revision of polymer network descriptions.

While some aspects of conventional theories are applicable to entangled and crosslinked semiflexible polymer networks [16,17,31], this study shows that different interactions between the constituting polymers should be accounted for more rigorously and included in the modeling of semiflexible polymer networks [17,19]. The experimental system used in this study has proven to be highly useful in experimental polymer physics before [16,17] and may be suitable for future research.

## Data Availability

We declare that the data supporting the findings of this study are available within the article.

## Abbreviations

The following abbreviations are used in this manuscript:

*n*HT	*n*-helix tube
wLX	weak LifeAct^®^-based crosslinker
MSD	mean squared displacement
